# Anticipation in the biosciences and the human right to science

**DOI:** 10.1093/jlb/lsae002

**Published:** 2024-02-19

**Authors:** Andrea Boggio

**Affiliations:** Department of Politics, Law and Society, Bryant University, 1150 Douglas Pike, Smithfield, RI 02917, United States

**Keywords:** human right to science, anticipation, anticipatory governance, bioscience futures, human gene editing, risk governance

## Abstract

Anticipation entails contemplating the beneficial and harmful impacts of scientific and technological progress. Anticipation has a long history in science, technology, and innovation policy partly due to future impacts of scientific progress being inescapable. The link between anticipation, an undertheorized concept, and human rights law is yet to be fully explored. This paper links anticipation to the rights to science, a lesser-studied human right codified in the International Covenant on Economic, Social, and Cultural Rights. The paper argues that the normative content of the right includes anticipation entitlements and duties. Combining the entitlements and duties with anticipation typologies leads to identifying three forms of anticipation that governments (and, in some cases, scientists) must carry out: beneficial, responsible, and participatory anticipation. The paper concludes by identifying three ways in which further conceptual work can enrich human-rights-based anticipation.

## I. INTRODUCTION

The pace of scientific and technological progress has rapidly accelerated in the past two decades. Advances in gene editing, synthetic biology, and artificial intelligence are transforming biosciences, both at the level of biomedical research and healthcare. While predicting where these and other technologies will eventually take the biosciences is speculative, benefits and harms of scientific progress can be anticipated. Anticipation is a set of ideas and practices connecting the future and the present. In general terms, it can be defined as the contemplation of future benefits and harms. Given the rapid pace of transformative innovation, anticipation is a necessity.

Debated for decades in science, technology, and innovation (STI) policy circles, anticipation has received little attention in international legal scholarship. This gap is troubling considering that international human rights law—particularly the human right to science codified in Article 15 of the International Covenant on Economic, Social, and Cultural Rights (ICESCR)—imposes on States the responsibility to prevent these emerging technologies from being used to violate human rights.

To fill this gap, I tackle the question of anticipation entitlements and duties in the normative context of the right to science. Largely neglected for decades after its codification, the right to science has been finally ‘awakened’ with the publication of the General Comment 25 by the Committee of Economic, Social, and Cultural Rights, a legal instrument providing authoritative guidance on its normative content. This ‘awakening’ offers scholars an opportunity to explore the relationship between, anticipation, the regulation of scientific progress and emerging technologies, and international law. In this vein, I analyze the relationship between anticipation and the right to science. My analysis shows that the normative content of the right includes the duty to contemplate future beneficial and harmful impacts of scientific progress and its applications and offers a trifold typology of anticipation responsibilities falling on the scientific community (responsible anticipation) and on governments (participatory and beneficial anticipation). The paper closes by highlighting the conceptual steps needed to further systematize and operationalize human-rights-based anticipation duties.

My interpretative approach is bottom-up, conceptualizing anticipation duties from the ground up, from the text of the ICESCR and key legal defining its normative content and defining human rights standards to which States are expected to adhere. Other scholars are engaged in top-down conceptual work, using the general principles of international law (prevention, precaution, and due diligence) as the departing point of their analysis.^1^ Moving from different premises, the two approaches are complementary, and both are needed to develop a robust understanding of human-rights-based anticipation. Exploring the link between the two ideas of anticipation is an opportunity to deepen our understanding of both.

After introducing the concept of anticipation in (Section II), I turn to the right to science, detailing the interpretive approach adopted in conceptualizing its normative content (Section III). Next, I identify anticipation entitlements and duties within the normative content of the right to science (Section IV) and discuss the three forms of anticipation required to realize the right to science (Sections V–VII). I conclude by identifying three ways in which further conceptual work can enrich human-rights-based anticipation and contribute to the diffusion of anticipation (Section VIII).

## II. DEFINING ANTICIPATION

Anticipation is a concept used in a variety of fields and with different meanings.^2^ On a very general level, anticipation is the *practice of contemplating future (beneficial and harmful) impacts of scientific progress and its applications*. Anticipation has been the object of inquiries in various disciplines.^3^ The intellectual roots of anticipation in the context of the study of STI can be traced to the development of future studies or *Futurologie*, a field founded by German philosopher Ossip Flechtheim. The basic premise of future studies is that the future must be contemplated to generate ideas to address pressing, contemporary social problems.^4^ According to early futurologists, the core of anticipation does not reside in forecasting the future but focuses on adapting and readapting our course of action in an effort to achieve a desirable future.

As future studies grew, this field developed a more robust idea of anticipation, framed as ‘an activity characterized by the use of a future representation (or a future scenario) (consciously or not) in order to guide actions in the present.’^5^ Anticipation centers around the idea that ‘the future is moving as it is consumed by the present, and it is also moving as the actors in the present change their visions about what they deem possible, likely, or desirable.’^6^ Futurists envision anticipation as a trigger to envision risks and benefits that have yet to materialize and then shape policy based on what was envisioned. In this light, anticipation became closely connected to foresight, which David Guston defines as ‘a methodologically pluralist approach to plausible futures with an emphasis on such methods as scenario development that provide a more diverse and normative vision compared with other methods that seek to identify a single, most likely future.’^7^

The study of anticipation has not been the intellectual monopoly of futurists or futures practitioners. It was picked up by scholars in science, technology, and society (STS) studies. A core premise of this body of scholarship is that science and technology are not only technically but also socially and politically constituted.^8^ In this light, anticipating science and technology futures is not the exclusive domain of those who possess technical expertise but is a shared, collective process^9^ that must be opened up to a wide range of inputs by creating new spaces of ‘public dialogue.’^10^ STS scholars have used anticipation to produce two frameworks: anticipatory governance and responsible innovation.^11^ Anticipatory governance fosters a dialog between actors with technical expertise (engineers, scientists, and humanists) and policy actors to increase mutual awareness, promote knowledge exchanges, and engage the reflective capabilities of governance mechanisms.^12^ Responsible innovation adopts a broader approach purporting to promote the social desirability of innovation by controlling and shaping innovation based on plausible scenarios. ^13^ To this end, the power of anticipation with inclusivity, reflexivity, and responsiveness.^14^

Further help in conceptualizing anticipation comes from two typologies of anticipation practices offered by scholars in recent years.^15^ The first is articulated Coutellec & Weil-Dubuc and picked up by Strub and Roche. The authors distinguish three forms of anticipation: predictive, adaptive, and projective.^16^*Predictive* anticipation strives to linearly predict the future based on probability calculation informed by the past (or the present) or based on ‘prophecy’ of the probable.^17^ Prediction or prophecy, Coutellec & Weill-Dubuc argue, ‘makes the future a mirror of the past.’^18^*Adaptive* anticipation assumes the future as radically open, focuses on the evolutionary and adaptive potential of individuals, societies, and institutions, and ‘mobiliz[es] of highly heterogeneous and transversal records of knowledge.’^19^ An unpredictable future must be anticipated through the lenses of epistemic diversity. *Projective* anticipation assumes that the future will be radically new, disrupted from any continuity with previous times, and deploys ‘a method for imagining the unimaginable, for thinking the unthinkable, to debate the impossible,’ leading to utopian rationality.^20^

A second typology is proposed by Spanish philosopher Sergio Urueña. The author identifies four approaches to anticipation: Predictivist, strategic, exploratory, and critical-hermeneutic. *Predictivist* approaches mirror the ‘predictive anticipation’ identified by Coutellec & Weill-Dubuc^21^*Strategic* anticipation posits a future as the ‘target of intended realisation or avoidance’^22^ and uses ‘causal chains, “driver forces” or roadmaps’ to generate a desirable outcome.^23^*Exploratory* anticipation breaks away from ‘traditional forecasting methods’^24^ and ‘opens up the plurality of plausible and desirable paths that could be considered within present socio-technical co-construction processes.’^25^ Finally, *critical-hermeneutic* approaches identify, understand, and criticize ‘the underlying epistemic and normative assumptions and the embedded meanings’ of future scenarios … to avoid the uncritical materialization of technological paths and co-production dynamics through uncritical (formal/informal) anticipations and deterministic ways of approaching the future.”^26^ Rather, this approach to anticipation strives ‘to renegotiate the meanings associated with futures and to emancipate actors from anticipatory power dynamics.’^27^

In the face of undertheorizing, the typologies, which offer ‘a more explicit characterization of intervention anticipations,’^28^ are useful to conceptualize and operationalize the link between anticipation and the right to science. It is time to turn to the human rights aspects of the analysis, beginning with laying out how to identify the normative content of the right to science and what that normative content is.

## III. INTERPRETING AND CONCEPTUALIZING THE RIGHT TO SCIENCE

The normative content of a human right includes entitlements and duties. What entitlements and duties fall under the right to science, and are anticipation entitlements and duties included in that account? To answer these questions, I shall first discuss how the normative content of a human right is identified and the specific interpretative challenges that the right to science presents.

The human right to science is a stand-alone, identifiable human.^29^ It is not well known outside a small but growing circle of scholars and practitioners leading a renaissance of interest in this right. Its roots predate the Universal Declaration of Human Rights. The first legal incarnation of the right can be dated back to 1947, when the Inter-American Juridical Committee, a committee of legal experts appointed to draft the American Declaration of the Rights and Duties of Man, adopted the ‘Anteproyecto de declaración de los derechos y deberes internacionales del hombre’ or ‘Preliminary Draft of a Declaration on the Rights and Duties of Men’ on December 31, 1945.^30^ Upon further revision, this document was adopted 2 years later, on December 8, 1947, and became the American Declaration.^31^ Since then, the human right to science has been included in the Universal Declaration of Human Rights and codified in the International Covenant on Economic, Social, and Cultural Rights, regional legal instruments, and a significant number of national constitutions.

Notwithstanding its long existence in international law, the right to science has been mostly neglected in human rights scholarship and practice. Until the late 2000s, the only major scholarly publication devoted to this right was the 2002 monograph by Richard Claude titled *Science in the Service of Human Rights*.^32^ While echoes of the right can be found in UNESCO standard-setting instruments of the late 1990s/early200s, the right was rediscovered when, under the auspices of UNESCO, a group of scholars and practitioners produced the *Venice Statement* in 2009.^33^ This instrument, with no binding force under international law, is the first organized attempt to spell out the normative content of the right. Most importantly, this triggered the interest of the UN human rights bodies and led to the appointment of Farida Shaheed as the first Special Rapporteur in the field of cultural rights. Shaheed produced the first official UN reports analyzing the right to science and its normative content. Her 2012 report was critical as it called for UN rights bodies to ‘review article 15 of the Covenant comprehensively, and envisage adopting a new general comment encompassing all rights recognized therein.’^34^ Shaheed’s recommendation was followed by the UN Committee on Economic, Social, and Cultural Rights (CESCR), which, after a long drafting and consultation process,^35^ adopted General Comment 25 on science and human rights in 2020.^36^ General Comment 25 represents a seminal moment in the renaissance of the right to science, not only because it legitimizes its invocation in human rights practice but also because it sets forth an authoritative interpretation of the right, which scholars can now use to forge their understanding of the normative content (entitlements and duties) of a right still very much undertheorized.

General Comment 25 is not the only basis for that theoretical work but a fundamental piece in the puzzle. General comments were initially intended to offer a systematic guide to States parties to the ICESCR in their efforts to monitor and report progress toward realizing the rights codified in the Covenant. The CESCR is expected to build on how states perceived their legal obligations under the ICESCR (known as ‘State practice’) regarding the particular right addressed in a general comment. This practice would emerge from the same progress reports that states must file periodically with the CESCR. However, when States Parties fail to report progress toward realizing a right, as with the right to science, State practice is difficult to ascertain and, therefore, an insufficient basis to guide the CESCR in drafting a general comment on that right. At this point, the options are two: waiting until a robust State practice emerges or empowering the CESCR to adopt general comments that identify the normative content and state obligation using a base different from State practice. General Comment 25 falls in this category. Its ultimate authoritativeness or normative legitimacy will be established only if, in subsequent State practice, States parties demonstrate to accept its interpretation of the right.^37^ These unfortunate circumstances, however, do not prevent legal scholars and practitioners from relying on an interpretative instrument that may not be authoritative. General Comment 25 and other ‘interpretative’ general comments aim to stimulate State practice. It is meant to be used in practice and relied upon as a source of the normative content of a human right. While provisional, its authoritativeness is real.

Based on these assumptions, the analysis presented in this paper relies greatly on General Comment 25 as a source of interpretation of the normative content of the right to science as codified in Article 15 of the ICESCR. However, this is not the only core legal instrument for interpreting the right to science. The standard-setting instruments adopted by UNESCO must also be considered critical to interpreting the right to science. These instruments, which typically are adopted by consensus of Member States and take the form of recommendations, declarations, and other nonbinding instruments, should be considered as evidence of State practice when setting standards linked to the normative content of the right to science.

UNESCO’s role in articulating international human rights standards is seldom recognized, but it is significant and paramount in the case of the right to science.^38^ Among the recommendations and declarations most relevant for a discussion of the right to science, one should mention the *2017 Recommendation on Science and Scientific Researchers*,^39^ the *2017 Recommendation of Ethical Principles in Relation to Climate Change*,^40^ the *2021 Recommendation on Open Science*,^41^ the 2021 *Recommendation on the Ethics of Artificial Intelligence*,^42^ and three declarations on the human genome, bioethics, and human rights: the *1997 Universal Declaration on Human Genome and Human Rights*,^43^ the *2003 International Declaration on Human Genetic Data*,^44^ and the *2005 Universal Declaration on Bioethics and Human Rights*.^45^

Based on this interpretive approach, I believe understanding the normative content of the right to science is facilitated by conceptualizing the right not as a single right but as an aggregation of rights, a ‘supercluster’ of rights.^46^ This conceptualization allows me to identify with sufficient specificity the entitlements and duties contained in the right to science so that I can then integrate anticipation in the analysis of the right. According to this view, the right to science contains four interrelated but distinct clusters of rights; each contains several discrete rights, which sometimes we break down, for the sake of clarity, in groups. The four clusters are the right to scientific progress, socially responsible science, participation in scientific progress, and benefit from scientific progress. The sub-cluster rights are 21 (see Table 1). Linking anticipation to the right to science requires exploring which of these clusters contained entitlements and duties that demand the contemplation of the future benefits and harms of scientific progress. Before getting there, I shall first address a more general question: is anticipation part of the normative content of the right to science?

**Table 1:** List of rights contained in the human right to science^a^

***Clusters of rights***	**Sub-cluster rights**
*I. Right to scientific progress*	Intellectual elements 1. Freedom of scientific thought 2. Freedom of scientific opinion and investigation 3. Freedom of scientific expression Social elements 4. Freedom of scientific assembly and association 5. Freedom of movement of scientists 6. Right of scientists to take part in the conduct of public affairs (passive and active participation). Labor elements 7. Right of scientists to work and follow scientific vocation freely 8. Right of scientists to just and favorable conditions of work and safe and healthy working conditions Cultural elements 9. right to take part in cultural life 10. right to benefit from the protection of the moral and material interests
*II. Right to socially responsible science*	11. Right to have scientific integrity respected 12. Right to responsible monitoring and anticipation of impacts
*III. Right to participate in scientific progress*	13. Right to scientific literacy 14. Right to access the scientific professions 15. Right to contribute to scientific progress (citizens as scientists and citizens as research subjects) 16. Right to participate in science policy affairs
*IV. Right to benefit from scientific progress*	17. Right to access scientific knowledge 18. Right to the anticipation and monitoring of the impact of applications of scientific progress Right to access existing applications of scientific progress 19. Right to the development of beneficial applications of scientific progress 20. Right to the diffusion of, and access to, beneficial applications of scientific progress 21. Right to policies aligned with scientific evidence

^a^Adapted from Andrea Boggio, *The Right to Participate In and Enjoy the Benefits of Scientific Progress and its Applications: A Conceptual Map*, 34 New York International Law Review 43–77 (2021).

## IV. ANTICIPATION RESPONSIBILITIES AND THE RIGHT TO SCIENCE

Having laid out the interpretative basis for identifying the normative content of the right to science, I now discuss whether these interpretative instruments consider ‘anticipation’ (ie the practice of contemplating future impacts of scientific progress) part of the normative content of the right to science. As a starting point, history must be acknowledged. The birth of the right to science as a human and the adoption of the instruments listed earlier took place in a vigorous debate over the benefits and harms of scientific progress. The advent of nuclear weapons and the Cold War intensely politicized the post-WWII discourse. This debate still informs the relationship between science and human rights. The drafters of both the UDHR and the ICESCR debated the relationship between human rights and the present and future consequences of scientific progress.^47^

While Article 15 of the ICESCR does not include language of anticipation, their echo permeates all instruments included in the ‘normative basis’ of the right to science. These instruments frame ‘anticipation’ in various ways and with different degrees of specificity. General Comment 25 places great importance on monitoring the beneficial and harmful impacts of research and applications on society, which is analyzed in the contest of conjunction with the participatory aspects of the right to science. The instrument identifies a ‘right to information and participation in controlling the risks involved in particular scientific processes and its applications,’ which engages scientists and governments in facilitating ‘public scrutiny and citizen participation’ in ‘decisions concerning the orientation of scientific research or the adoption of certain technical advancements’.^48^ Additional language in General Comment 25 reinforces the claim that the normative content of the right to science comprises anticipation. For instance, the instrument states, ‘States parties have to adopt policies and measures that expand the benefits of these new technologies while simultaneously reducing their risks’.^49^ The need to consider scientific progress’s impacts is also mentioned in the sections discussing the ‘special protection for specific groups.’^50^

The contemplation of the impacts of scientific progress permeates the UNESCO standard-setting instruments. The *Recommendation on Science and Scientific Researchers*, the right to science instrument addressing scientists’ duties more directly, parses this responsibility into various dimensions. Contemplation of impacts relates to the scientists’ ‘ability to review a problem or situation in perspective and in proportion, with all its human implications’,^51^ possessing the ‘skill’ of ‘isolating the civic and ethical implications, in issues involving the search for new knowledge and which may, at first sight, seem to be technical only’,^52^ engage in ‘vigilance as to the probable and possible social and ecological consequences of research and development activities’,^53^ and ‘willingness to communicate with others not only in scientific and technological circles but also outside those circles’.^54^ Furthermore, ‘researchers should seek to minimize impacts on living subjects of research and on the natural environment and should be aware of the need to manage resources efficiently and sustainably’.^55^ Finally, governments must support initiatives concerning.

“(a) the impact of science on future generations;

(b) the interconnection between various forms of life;

(c) the role and responsibility of human beings in the protection of the environment, the biosphere and biodiversity.”^56^

Impacts of scientific progress feature prominently in the *Declaration of Ethical Principles in Relation to Climate Change*, where ‘impact’ appears 37 times. As a framework for all actors involved in the life cycle of AI systems, the Declaration calls for ‘dealing responsibly with the known and unknown impacts of AI technologies on human beings, societies, and the environment and ecosystems’^57^ and for ‘the continuous assessment of the human, social, cultural, economic and environmental impact of AI technologies’.^58^ The actors involved in the life cycle of AI systems are also expected to participate in the ethical impact assessment and other processes or tools to monitor the impact of AI technologies on society.^59^ ‘[E]enhancing the societal impact of science and increasing the capacity of society as a whole to solve complex interconnected problems’ is a guiding principle of the *Recommendation on Open Science*.^60^ Finally, the impacts of progress in genetics and genomics animated the three instruments addressing bioethical themes,^61^ even though these instruments do not expressly address the responsibilities of scientists regarding impacts.

For completeness, it is essential to acknowledge that anticipation entitlements and duties can also be traced to more general duties recognized under international law, namely the duty to prevent, the precautionary principle, and due diligence.^62^ General Comment 25 incorporates due diligence considerations, stating, ‘States parties should establish a legal framework that imposes on non-State actors a duty of human rights due diligence, especially in the case of big technology companies.’^63^ I address the relationship between precaution and anticipation in the last section of the paper. While important to articulate the full extent to which anticipation is recognized in international law, engaging fully with these concepts is not vital to the argument presented in this paper. My argument is more circumspect, focusing exclusively on the normative content of the right to science as ascertained from a discreet range of instruments centering on science and human rights.^64^

Having established that the normative content of the right to science contains anticipation entitlements and duties, my attention now focuses on specifying what forms anticipation takes within the right to science framework. As discussed earlier, this analysis is facilitated by conceptualizing the right to science as a supercluster of rights comprising the right to scientific progress, responsible scientific progress, participation in scientific progress, and beneficial scientific progress. Based on this conceptualization, anticipation can be linked to all four rights leading to identify three forms of anticipation that must be carried out to realize the right to science: beneficial anticipation, responsible anticipation, and participatory anticipation (Table 2).

**Table 2:** Matrix of anticipation entitlements/duties under the right to science

		**… anticipation**
*Right to …*	*benefit from scientific progress*	beneficial
	*scientific progress*	responsible
	*socially responsible science*	
	*participate in scientific progress*	participatory

## V. BENEFICIAL ANTICIPATION

*Beneficial anticipation* is normatively connected to the *right to benefit from scientific progress*, which includes the right to access the ‘material results from the applications of scientific research’ (vaccinations, fertilizers, technological instruments, and the like), ‘scientific knowledge and information’ and the ability to of ‘forming critical and responsible citizens who are able to participate fully in a democratic society.’^65^ It ensures access to scientific knowledge and technology (existing applications of scientific progress), the development of new applications of scientific progress, and policies based on scientific evidence.^66^ Beneficial anticipation focuses on the substance of anticipation. It requires setting up processes to determine the conditions under which scientific progress is possible in the present in relation to future impacts.

Traditional *ex-ante* approaches (ie risk assessment practices and precautionary approaches) used to assess the benefits and harms of scientific and technological progress typify beneficial anticipation. They rely on the assumption that predictions are ‘expected to provide accurate knowledge regarding the probable impacts that a technology could produce if implemented.’^67^ ‘Ideally,’ the National Research Council of the National Academies notes, ‘the design of a risk assessment takes into account foreseeable consequences of decisions, including substitution risks … side effects of risk controls, … and other potential adverse outcomes associated with decisions.’^68^ Strategic forms of beneficial anticipation are widely used for designing and assessing R&D policies and/or research agendas. These include ‘“pull” and “push” innovation strategies towards pre-settled desired target futures (see, for example, regarding nanotechnology).’^69^

Drug approval is an exemplar of predictive anticipation in the biosciences. This process enables the government to approve the marketing of a new drug based on preclinical and clinical data showing that the drug benefits patients and that these benefits outweigh the known related risks (ie side effects). A determination that the benefits outweigh the risks and that a quality product can be manufactured, the new drug is approved. In doing so, the government relies on inductive reasoning that the data presented can be extrapolated to future patients. This data must indicate that the balance between benefits and harms will remain predictably the same in the future. Mainstream risk assessment of drugs typically incorporates some consideration of future contingents in the form of post-market surveillance of approved drugs. Monitoring the safety of a drug after approval for marketing permits the adjustment of decisions based on predictive anticipation based on the emergence of new data begging for a refinement of earlier predictions.

Current praxes in beneficial anticipation are critiqued on account of an excess of caution and reliance on technocratic knowledge and democratic deficit.^70^ The first line of criticism questions the adequacy of these practices to satisfy beneficial anticipation standards. Excessive caution is often linked to the misuse of the *precautionary principle*. This principle prescribes ‘take no risky action’ under circumstances of uncertainty concerning risks presenting specific characteristics.^71^ The concept of ‘no risky action’ typically results in precautionary restrictions, ranging from tight regulation to full bans.^72^ The principle is generally accepted in international law and endorsed by General Comment 25 as compatible with the right to science.^73^ It is worth noting two points without entering into the debate on the merits of a precautionary approach. First, a close reading of General Comments No. 25 reveals that the precautionary principle is hereto identified as one of the tools available to governments to engage in anticipation of risks and benefits, not as the exclusive tool.^74^ Second, applying the precautionary principle must be consistent with the human rights framework. It can lead to restriction of scientific freedom and access to scientific progress and its applications only if the limitations standards codified in Article 4 of the ICESCR are met. Accordingly, restrictions adopted as a result of precautionary anticipation must be necessary and proportionate. Necessity requires that the precautionary goal that a prohibition of scientific activities aims to achieve cannot be reached by a less restrictive measure (for instance, by regulatory oversight).^75^

The second line of criticism calls into play participatory anticipation standards. Efforts to address the gap in non-technical participation risk assessment have been increasingly made in recent years. Examples can be found in programs involving patients in post-marketing surveillance. As van Hoof and colleagues point out, patients ‘can detect medication errors, quality issues, drug misuse and other important drug safety concerns and play an active role in the therapeutic decision-making process.’^76^ For example, they note that ‘during the coronavirus disease 2019 (COVID-19) pandemic, there has been a surge in the number of reports from the general public regarding COVID-19 vaccines, which can contribute to our knowledge of the safety profile of these vaccines in daily practice.’^77^ Patient advocacy in rare diseases has enriched participatory anticipation, impacting research priority setting, research protocol design, and patient recruitment in clinical trials. Through participatory practices, patients can stir therapeutic innovation in a direction that is more inclusive of patients with rare diseases and, more generally, of *more* patients. The anticipatory impacts of these initiatives, however, remain unclear.^78^

## VI. RESPONSIBLE ANTICIPATION

*Responsible anticipation* is an entitlement/duty that scientists contemplate future impacts of scientific progress as part of their scientific activities.^79^ It requires the scientific community’s engagement in a self-directed, collective exercise of contemplating future impacts of scientific progress. Normatively, it emerges from the combination of the right to scientific progress and the right to responsible scientific progress. The *right to scientific progress* is an entitlement ensuring that ‘scientific progress happens.’^80^ It is a right of society to scientific progress, and it can be realized (only) when scientists enjoy scientific freedom. The *right to socially responsible science* is everyone’s right that scientists act responsibly. It stems from scientific freedom and the duty of scientists to enjoy it responsibly. These two rights are two sides of the same coin—one representing scientists’ rights and the other representing their duties—and therefore, the contemplation of the benefits and harms of scientific progress is carried out by scientists as a matter of right and duty.^81^

Right-to-science scholars consider scientific responsibility a constitutive element of the normative content of this human right. In her analysis of Article 15(1)(b) of the ICESCR, Helle Porsdam argues that ‘[i]n a democratic society, scientific research can never be entirely free. It must always be conducted in a socially and ethically responsible manner.’^82^ While not supported by either the text of Article 15 of the ICESCR or General Comment 25, support for considering scientific responsibility constitutive of the human right to science comes from an evolutionary interpretation of the right. The uncontroversial realization of the normative link between scientific freedom and responsibility is relatively recent, and subsequent to the drafting and adoption of the ICESCR.^83^ Based on a survey of ‘official documents and structures of the scientific community,’^84^ Heather Douglas concluded that since 2010 scientific responsibility has been seen ‘as a necessary partner to scientific freedom, as yoked to freedom.’^85^ ‘No longer was freedom to set research agendas thought to be only secured when one also was free from social responsibility for the impacts of research,’ Douglas adds.^86^ Scientific responsibility includes, among other things,^87^ a duty to ‘bear[] responsibility for the impacts of research’^88^ and ensure that innovation is ‘embedded in social context to anticipate and respond to arising ethical and societal concerns.’^89^ Furthermore, scientific freedom permeates recent UNESCO standard-setting instruments.^90^ Standards concerning the anticipatory aspects of scientific responsibility are embedded in the *Recommendation on Science and Scientific Researchers*, which recommends that scientists engage in ‘vigilance as to the probable and possible social and ecological consequences of research and development activities’^91^ with a ‘willingness to communicate with others not only in scientific and technological circles but also outside those circles.’^92^ Researchers are also expected to ‘seek to minimize impacts on living subjects of research and on the natural environment and should be aware of the need to manage resources efficiently and sustainably.’^93^

An early example of a predictive practice of responsible anticipation that relied heavily on considerations of precautions is the Asilomar Conference on recombinant DNA.^94^ 140 professionals, mostly biologists, convened in 1975 to discuss the future of research and applications of the then-novel technology.^95^ An outcome of the Conference was the adoption of voluntary guidelines reaffirming the freedom to conduct basic research on recombinant DNA, with appropriate safeguards, while calling for ‘a worldwide moratorium on the work, followed by an international conference of experts at which the nature and magnitude of the risks could be assessed.’^96^

A more recent example is the convening of the International Summit on Human Gene Editing, an initiative supported by the Royal Society, the UK Academy of Medical Sciences, the US National Academies of Sciences and Medicine, and the World Academy of Sciences.^97^ Guided by pragmatism and precautionary thinking, the Summit has engaged in strategic anticipation defining pathways for future applications of human gene editing. Since its first meeting in 2015, the Summit has approached gene editing predictively and precautionarily with consideration to the impact of scientific progress on future generations. Notably, the 2023 Summit reached a conclusion on germline gene editing that is more precautionary than the 2018 conclusion. While in 2018, the Summit concluded that ‘it is time to define a rigorous, responsible translational pathway toward such trials,’^98^ in 2023, the conclusion is that:

Preclinical evidence for the safety and efficacy of heritable human genome editing has not been established, nor has societal discussion and policy debate been concluded. (In some cases, preimplantation genetic testing is among the alternatives.) Heritable human genome editing should not be used unless, at a minimum, it meets reasonable standards for safety and efficacy, is legally sanctioned, and has been developed and tested under a system of rigorous oversight that is subject to responsible governance. At this time, these conditions have not been met.^99^

The Summit’s posture is technocratic, relying almost exclusively on scientific evidence. The reasoning is linear, drawing a line from today’s knowledge and practices into a future, mixing predictive and strategic considerations. Recurring meetings permit adjustment of the anticipatory conclusions of the previous meeting. At the same time, the Summit has called for an ‘ongoing forum’ tasked with developing shared norms of how to proceed.^100^

These are some examples of responsible anticipation, a collective duty of scientists, which must be discharged as a scientific community for the scientific community. When engaging in responsible anticipation conversations, the scientific community must enjoy some degree of autonomy, recognized, and protected in Article 15 of the ICESCR as ‘freedom indispensable for scientific research.’ Governments sit on the sidelines, playing a limited role. They have a responsibility to ensure that scientists are free to engage in responsible anticipation and do engage in responsible anticipation. Governments must guarantee scientists’ ability to associate, meet, travel, express their views, and access adequate support (funding, training, expertise) to take part in responsible anticipation. Governments must also ensure that scientists are not prevented from communicating their assessments to the public. This entitlement is part of the right to participate in public affairs as codified in Article 25 of the ICCPR and incorporated in the notion of scientific freedom.

While Asilomar and similar responsible anticipation initiatives can be criticized for their insularity and technocratic posture,^101^ one must also recognize that, to be effective, responsible anticipation must be carried out with some detachment from society. This is not to say that scientists must not engage in broader anticipatory conversation. On the contrary, they have a duty to engage in responsible anticipation and share the viewpoints resulting from responsible anticipation initiatives with policymakers and the general public. The duty of the scientific community to engage with society does not exclude the right to engage in responsible anticipation autonomously. It reinforces it. There is value in reflections on the potential impact of scientific progress driven by technical expertise. This way, scientists can focus on the impact of their work and deliberation based only on considerations under their control. Insularity from society does not mean governments must follow responsible anticipation interventions’ recommendations or that the conclusions proposed by scientists must be accepted by society.

On the contrary, these interventions must be scrutinized, debated, and possibly rejected. In their anticipatory praxis, governments cannot ignore the evidence but reach policy conclusions different from those of the scientific community. According to General Comment 25, ‘government policies and programmes’ must be ‘aligned’ ‘with the best available, generally accepted scientific evidence.’^102^ However, they may depart from the conclusions of the scientific community. In spelling out these standards, General Comment 25 uses the term ‘evidence’ in contrast to other sections of the instrument which refer to ‘scientific knowledge.’

No matter how responsible anticipation conclusions are received in broader societal debates, the voice of scientists, acting autonomously and internally, is a critical piece of the puzzle because of the unique perspective these initiatives bring to the societal conversation. Scientists engaging with an audience of non-specialists face a climate in which science is politicized. Intervening in politically polarized debates, scientists are pressured to take strong stances to defend their position. Responsible anticipation is an opportunity for the scientific community to develop a viewpoint that can be used as an anchor for scientists to manage the pressure of political polarization when shared with broader audiences.

## VII. PARTICIPATORY ANTICIPATION

*Participatory anticipation* is a form of anticipation is grounded on the *right to participate in scientific progress*, that is, ‘the right of every person to take part in scientific progress and in decisions concerning its direction.’^103^ This right can be parsed into two entitlements: a right to scientific literacy and a right to participate in science policy decision-making. Accordingly, the public is entitled to acquire sufficient scientific literacy to understand scientific knowledge to meaningfully contribute to debates and decisions concerning the impacts of scientific progress. It is also an entitlement to actively participate in public affairs (debates and decisions) concerning the impacts of scientific progress. It is not an entitlement to dictate what scientists should or should not do but to participate in decisions that may shape the direction of science and, under exceptional circumstances, limit scientific freedom provided the limitations meet the standards set forth in Article 4 of the International Covenant on Civil and Political Rights (ICCPR) and the ICESCR. The ICCPR matters in this context because the right to participate in scientific affairs springs from the convergence of the right to science with the right to participate in political and public affairs codified in Article 25 of the ICCPR.

To carry out participatory anticipation in line with human rights standards, governments must create opportunities for the public to engage actively in STI policy. According to General Comment 25, ‘participation’ in scientific affairs encompasses the active contribution of citizens to scientific progress.^104^ As such, it implicates using ‘inclusive methodologies of consultation and deliberative techniques to engage wide-ranging stakeholders to embed technologies with social and moral value.’^105^ The types of interventions that can be implemented to enable the public to actively engage in STI policy fall into two categories: *public consultation*, and *participation*.

In *public consultation*, ‘information is conveyed from members of the public to the sponsors of the initiative, following a process initiated by the sponsor … The information elicited from the public is believed to represent currently held opinions on the topic in question.’^106^ Public consultation methods include citizens’ panels, consultation documents, internet-based consultations, focus groups, open spaces, opinion polls, referenda, study circles, surveys, telepolling, and televoting.

*Public participation* methods involve an exchange between members of the public and the sponsors of the initiative. Participation entails:

some degree of dialogue in the process that takes place (usually in a group setting), which may involve representatives of both parties in different proportions (depending on the mechanism concerned) or, indeed, only representatives of the public who receive additional information from the sponsors prior to responding. Rather than simple, raw opinions being conveyed to the sponsors, the act of dialogue and negotiation serves to transform opinions in the members of both parties (sponsors and public participants).^107^

In some cases, these processes include an expert-mediated deliberation component, ‘a space in which “long-held views of a pluralistic society may be revised through contestatory civic engagement” that facilitates the development of an “enhanced state of mutual understanding”.’^108^ Public participation methods include action planning workshops, citizen juries, consensus conferences, deliberative opinion polls, negotiated rulemaking, planning cells, task forces, and town meetings with voting.

An example of effective participatory anticipation is the public consultation conducted by WHO’s Expert Advisory Committee on Developing Global Standards for Governance and Oversight of Human Genome Editing in 2020. Its ‘Call for contribution’ invited input from anyone with no restrictions of expertise or qualifications and included this statement of intent:

In an effort to understand and include a wider set of views, the Committee has provided a draft of its governance framework for public comment. The Committee are interested in views on the approach they have taken to the governance framework, its content, as well as insights on good governance of human genome editing.^109^

The Call included a survey^110^ distributed to the public asking questions about a Draft Framework for Governance, published on the WHO website.^111^ Notably, the Draft included seven scenarios presenting a ‘narrative description of a possible future event[,] relevant components from the governance framework … including … values, principles and goals [and] questions to be considered.’^112^ This strategy presents the opportunity to solicit feedback on imagined futures rather than predictions. Disappointingly, though, the survey did ask specific questions about these scenarios, which the Draft presented as tools to guide the use of the Draft. The potential of these scenarios as a tool for anticipatory participation was thus not truly deployed because they were not used to raise questions about the Draft and its assumptions. Instead, it directs readers on how to use it when confronted with plausible futures.

Civil society can also initiate participatory anticipation.^113^ Examples of gene editing include the ARRIGE (Association for Responsible Research and Innovation in Genome Editing) Initiative^114^ and the Global Observatory for Genome Editing.^115^ A proposed, yet-to-be-implemented model is a global citizens’ assembly.^116^ This assembly would operate a non-governmental platform, ideally funded by public money, where randomly selected members of the public from various regions of the world participate in a public engagement exercise. The assembly ‘would meet over a week or more, hear presentations from experts and advocates, deliberate among themselves in small groups (each of which would have a facilitator) then in plenary session, and produce a report that summarized key concerns and recommendations.’^117^ An advisory committee would help members navigate the information ‘ensuring that participants receive balanced information.’^118^

In sum, participatory anticipation entitles the public to have a meaningful opportunity to contribute to shaping science policy by taking part in consultative and participatory processes contemplating future impacts of scientific progress in the long-term (whether germline editing should be authorized) and in the short-term (whether a specific treatment should be made available to a specific population).

## VIII. TOWARD HUMAN-RIGHTS-ORIENTED ANTICIPATION PRAXES IN THE BIOSCIENCES

In this paper, I have explored the links between anticipation and the human right to science. The normative content of this right includes a responsibility to contemplate present and future benefits and harms of scientific progress and its applications. As a human rights obligation, anticipation can be organized in three forms: beneficial, participatory, and responsible anticipation. The first two fall on the shoulders of States, the latter on the shoulders of the scientific community, with States owing secondary duties to respect, protect, and fulfill scientists’ self-governance powers.

Theoretical work is still needed to fix a framework that fully operationalizes human-rights-based anticipation. Four strands of work will advance this field. First, a more robust account of how the anticipation responsibilities based on the right to science relate to the more general responsibilities imposed by international law. This work requires connecting each form of anticipation with the duty to prevent violations of human rights, the precautionary principle, and due diligence. These connections with general obligations will enrich how right-specific obligations should be construed and operationalized.^119^

In this vein, it is also critical that the connection between anticipation praxes and broader questions of justice and redistribution are explored. This work must address the ability of these praxes to satisfy nondiscrimination, a principle at the cornerstone of human rights law, and ensure progress for everyone.

Third, theoretical work must identify human-rights standards and anticipation praxes to be assessed against them. Pointing to the existence of anticipation obligations, as I do in this paper, is necessary but insufficient. Further work must spell out precise standards for operationalizing these obligations into practice. These standards should then be used to measure adherence of existing and prospective anticipatory practices to human rights expectations. I have briefly surveyed anticipation praxes in the biosciences, emphasizing gene editing governance. A more robust empirical account of these practices would help clarify further the extent to which anticipation is already practiced and the standards that inform current anticipation mechanisms. This exercise must include a variety of anticipation activities, ranging from well-established praxes to more novel and disruptive approaches, that are (or could be) implemented and must adhere to human rights standards to be kept and replicated. If they fall short, they must be adjusted or discarded.

Finally, indicators must be developed to assess adherence to human rights standards. These indicators should measure the extent to which States have created an enabling environment for beneficial and participatory anticipation and respect the autonomy of the scientific community to engage in responsible anticipation. Indicators play important functions in the international human rights system.^120^ They serve as tools to monitor States Parties’ compliance with treaty obligations. They also serve as benchmarks for States and nonstate actors (including the scientific community) to prioritize actions that foster the respect and realization of human rights. Finally, they enable activists to expose shortcomings in anticipation practice and advocate for change.

As Jerome Ravetz argued,^121^ effective anticipation requires asking ‘what if’ questions, that is, ‘about what is known, what is likely, what is plausible, and what is possible.’^122^ More work needs to be done to identify how policymakers, scientists, and the public can be engaged in the full range of questions concerning the future of scientific and technological progress. The human right to science serves as an ideal anchor of national and international practices aiming to anticipate the benefits and harms of scientific progress and its applications.

## Footnotes

1 See the papers soon to be published in a special issue of the *International Journal of Human Rights*.

2 See, Roberto Poli, *Understanding the Future*, *in*Introduction to Anticipation Studies 59, 73–74 (Roberto Poli ed., 2017), https://doi.org/10.1007/978-3-319-63023-6_4 (last visited Feb. 15, 2023) (exposing the need for a well-developed, cohesive theory of anticipation, which would combine the various strands of anticipation approaches).

3 Poli, *supra* note 2, at 6 (locating anticipation in the natural sciences, human and social sciences, and philosophy).

4 Ossip K. Flechtheim, *Teaching the Future*, 16 J. High. Educ. 460 (1945). Flechtheim was concerned with problems such as preventing new wars, stabilizing population growth, ending poverty and exploitation, and fostering sustainability.

5 Sergio Urueña, *Responsibility through Anticipation? The ‘Future Talk’ and the Quest for Plausibility in the Governance of Emerging Technologies*, 15 NanoEthics 271, 277 (2021).

6 Markus S Schulz, *Future moves: Forward-oriented studies of culture, society, and technology*, 63 Curr. Sociol. 129, 129 (2015).

7 David H. Guston, *Understanding ‘anticipatory governance,’* 44 Soc. Stud. Sci. 218, 226 (2013).

8
Langdon Winner, Autonomous technology: Technics-out-of-control as a theme in political thought (1978).

9 Carl Mitcham, *Co-responsibility for research integrity*, 9 Sci. Eng. Ethics 273 (2003).

10 Alan Irwin, *The Politics of Talk: Coming to Terms with the ‘New’ Scientific Governance*, 36 Soc. Stud. Sci. 299, 300–303 (2006). The analysis of anticipation intersects with the concept of sociotechnical imaginaries, developed in the STS literature, as a framework to investigate how visions of the future drive perceptions of the risks or benefits of scientific advancements. *See*, Sheila Jasanoff and Sang-Hyun Kim, Dreamscapes of modernity: Sociotechnical imaginaries and the fabrication of power (2015).

11 Urueña, *supra* note 5, at 271–273.

12
*Id.* at 276.

13 Jack Stilgoe, Richard Owen & Phil Macnaghten, *Developing a framework for responsible innovation*, 42 Res. Policy 1568 (2013).

14 René von Schomberg, *Prospects for technology assessment in a framework of responsible research and innovation*, *in*Technikfolgen abschätzen lehren: Bildungspotenziale transdisziplinärer Methoden 39 (Marc Dusseldorp & Richard Beecroft eds., 2012), https://doi.org/10.1007/978-3-531-93468-6_2 (last visited Feb. 16, 2023).

15 Jean-Daniel Strub & Johan Rochel, *10. Ethics of anticipation*, GESDA (2020), https://gesda.global/wp-content/uploads/2020/11/GESDA-SAB-10_Ethics-of-anticipation.pdf; Léo Coutellec & Paul-Loup Weil-Dubuc, *Les figures de l’anticipation. Ou comment prendre soin du futur*, 2 Rev. Fr. Déthique Appliquée 14 (2016); Urueña, *supra* note 5, at 277–287.

16 Strub and Rochel, *supra* note 15; Coutellec and Weil-Dubuc, *supra* note 15, at 15.

17 Coutellec and Weil-Dubuc, *supra* note 15, at 15.

18
*Id.* at 15.

19
*Id.* (‘Unknowns and uncertainties are neither ignored nor reduced in a risk calculation, they are at the heart of knowledge production devices’).

20
*Id.* at 16.

21 Urueña, *supra* note 5, at 279.

22
*Id.* at 283.

23
*Id.*

24
*Id.* at 284.

25
*Id.*

26
*Id.* at 286.

27
*Id.* While Urueña does not explicitly discuss the determinants of anticipatory power dynamics, these likely include the concepts of race, gender, class, influence, and privilege as they are widely used in social sciences analysis of power and justice.

28
*Id.* at 277.

29 Mikel Mancisidor, a legal scholar and member of the CESCR, persuasively argues that, on practical and conceptual grounds, we should use the expression ‘right to science’ rather than its clucky and descriptively incomplete permutations such as ‘right to share in scientific advancement and its benefits’ (Universal Declaration on Human Rights), ‘right to enjoy the benefits of scientific progress and its applications’ (ICESCR), or ‘right to participate in and to enjoy the benefits of scientific progress and its applications’ (CESCR, General Comment 25). See, Mikel Mancisidor, *The Dawning of a Right: Science and the Universal Declaration of Human Rights (1941–1948)*, *in*The Right to Science 17 (Helle Porsdam & Sebastian Porsdam Mann eds., 1 ed. 2021), https://www.cambridge.org/core/product/identifier/9781108776301%23CN-bp-1/type/book_part (last visited Aug. 19, 2022).

30 Comité Jurídico Interamericano, ‘Anteproyecto de declaración de los derechos y deberes internacionales del hombre, Rio de Janeiro, 31 diciembre 1945’, in Comité Jurídico Interamericano, *Recomendaciones e informes, Documentos Oficiales (1945–1947)*, Departamento de Imprensa Nacional, Rio de Janeiro, 1950, pp. 49–59. For the commentary, see ‘Informe anexo al anteproyecto de declaración de los derechos y deberes internacionales del hombre’, *ibid.*, pp. 61–115. The English version of the Draft Declaration, including a commentary, can be found in Pan American Union, *Draft Declaration of the International Rights and Duties of Man and Accompanying Report, Formulated by the Inter-American Juridical Committee in accordance with Resolutions IX and XL of the Inter-American Conference on Problems of War and Peace held at Mexico City, February 21–March 8, 1945*, Pan American Union, Washington DC, March 1946.

31 Comité Jurídico Interamericano, *Declaración de los derechos y deberes internacionales del hombre*, *Rio de Janeiro, 8 diciembre 1947*, in Comité Jurídico Interamericano, *Recomendaciones e informes, Documentos Oficiales (1945–1947)*, pp. 185–193. The English version of the second draft can be found in Thomas Buergenthal and Robert E. Norris, eds. *Human Rights: The Inter-American System*, Oceana Publications, Part I, Chapter IV, p. 9 (Section C).

32
Richard Pierre Claude, Science in the service of human rights (2002).

33 UNESCO, *The Right to enjoy the benefits of scientific progress and its applications (Venice Statement)*, UNESCO (2009), https://unesdoc.unesco.org/ark:/48223/pf0000185558 (last visited Dec. 3, 2021).

34 UN Human Rights Council, *Report of the Special Rapporteur in the field of cultural rights, Farida Shaheed: The right to enjoy the benefits of scientific progress and its applications*, A/HRC/20/26 (May 14, 2012).

35 I was invited by the CESCR to give testimony on a draft of the general comment on the day of general discussion, which was held in Geneva in October 2018.

36 UN Committee on Economic, Social and Cultural Rights, General comment No. 25 on Science and economic, social and cultural rights Art. 15.1.b, 15.2, 15.3, and 15.4, E/C.12/GC/25, (2020), para. 11 (hereinafter, ‘General Comment 25’).

37 Andrew Mazibrada, Monika Plozza & Sebastian Porsdam Mann, ‘Innovating in Uncharted Terrain: On Interpretation and Normative Legitimacy in the CESCR’s General Comment No. 25 on the Right to Science,’ The International Journal of Human Rights, 14–17 (2023), https://doi.org/10.1080/13642987.2023.2234298.

38 Yvonne Donders & Konstantinos Tararas, *Mainstreaming Science and Human Rights in UNESCO*, *in*The Right to Science: Then and Now 124, 124–139 (Helle Porsdam & Sebastian Porsdam Mann eds., 2021), https://www.cambridge.org/core/books/right-to-science/mainstreaming-science-and-human-rights-in-unesco/28FF4E72961930AE0C51B23BEAB392A6 (last visited Feb. 9, 2023).

39 UNESCO, *Recommendation on Science and Scientific Researchers*, adopted at the 39th session of the General Conference on 13 November 2017, 39 C/Resolutions, https://unesdoc.unesco.org/ark:/48223/pf0000260889.page=116 (last visited Feb. 9, 2023).

40 UNESCO, *Declaration of Ethical Principles in Relation to Climate Change*, adopted at the 39th session of the General Conference on 13 November 2017, SHS/BIO/PI/2017/2, https://unesdoc.unesco.org/ark:/48223/pf0000260889.page=127, (accessed Feb. 9, 2023).

41 UNESCO, *Recommendation on Open Science*, adopted by the General Conference of UNESCO at its 41st session, on 23 November 2021, SC-PCB-SPP/2021/OS/UROS, https://unesdoc.unesco.org/ark:/48223/pf0000379949.locale=en (accessed Feb. 9, 2023).

42 UNESCO, *Recommendation on the Ethics of Artificial Intelligence*, adopted at the 41st session of the General Conference on 24 November 2021, SHS/BIO/PI/2021/1, https://unesdoc.unesco.org/ark:/48223/pf0000381137, (accessed Mar. 12, 2023).

43 UNESCO, *Universal Declaration on the Human Genome and Human Rights*, adopted at the 29th Session of the General Conference on 11 November 1997, BR/2001/PI/H/1, https://en.unesco.org/themes/ethics-science-and-technology/human-genome-and-human-rights (accessed Mar. 12, 2023). The 1997 Declaration was subsequently endorsed by the United Nations General Assembly with Resolution AIRES/53/152 on 9 December 1998 at its 53rd session.

44 UNESCO, *International Bioethics Committee, Human Genetic Data: Preliminary Study by the IBC on its Collection, Processing, Storage and Use* (SHS-503/01/CIB-8/3 (Rev.2), 15 May 2002), https://unesdoc.unesco.org/ark:/48223/pf0000123522, (accessed Mar. 12, 2023).

45 UNESCO, *Universal Declaration on Bioethics and Human Rights*, adopted by the General Conference on 19 October 2005, 33 C/Resolutions, p. 74, https://en.unesco.org/themes/ethics-science-and-technology/bioethics-and-human-rights (accessed Mar. 12, 2023).

46 Andrea Boggio, *The Right to Participate In and Enjoy the Benefits of Scientific Progress and its Applications: A Conceptual Map*, 34 N. Y. Int. Law Rev. 43 (2021); Cesare P.R. Romano & Andrea Boggio, The Human Right to Science. History and Evolution (2024). Our terminology borrows the language of astronomy, where the term ‘supercluster’ describes a large group of smaller galaxy clusters, each containing thousands of galaxies and millions of stars.

47 Mancisidor, *supra* note 29, at 22–23; Helle Porsdam, Science as a Cultural Human Right 50–52 (2022).

48 General Comment 25, para. 55.

49
*Ibid*., para. 74.

50
*Ibid*., paras. 32 (women) and 40 (traditional knowledge and indigenous peoples).

51 Recommendation on Science and Scientific Researchers, *supra* note 14, para. 14(d)(iii).

52
*Ibid*., para. 14(d)(iv).

53
*Ibid*., para. 14(d)(v).

54
*Ibid*., para. 14(d)(vi).

55
*Ibid*., para. 16(a)(ii).

56
*Ibid*., para. 19.

57 Declaration of Ethical Principles in Relation to Climate Change, *supra* note 39, para 1.

58
*Ibid*., para 31.

59
*Ibid*., paras 50–70.

60 Recommendation on Open Science, *supra* note 14, para 14.a.

61 See, eg UNESCO, *Universal Declaration on the Human Genome and Human Rights*, *supra* note 41, Arts. 6 and 12; UNESCO, *International Declaration on Human Genetic Data*, *supra* note 42, Arts. 4 and 6; UNESCO, *Universal Declaration on Bioethics and Human Rights*, *supra* note 43, Arts. 4, 11, and 16–17.

62 Samantha Besson, ‘La Due Diligence En Droit International (Volume 409)’, *Collected Courses of the Hague Academy of International Law*, http://dx.doi.org/10.1163/1875-8096_pplrdc_A9789004445055_02 (last accessed Mar. 12, 2023) (defining due diligence as an obligation ‘to minimise risks to human rights no matter the source of the risks, but without disproportionately limiting individual freedom or State sovereignty’).

63 General Comment 25, para. 75.

64 For a discussion of anticipation, the right to science, and the general principles of international law, see a forthcoming special issue of the *International Journal of Human Rights*.

65 General Comment 25, para. 8.

66 Boggio, *supra* note 46, at 70–72.

67 Urueña, *supra* note 5, at 279.

68
National Research Council of the National Academies, Science and Decisions: Advancing Risk Assessment 72–73 (2008), https://nap.nationalacademies.org/catalog/12209/science-and-decisions-advancing-risk-assessment (last visited Aug. 18, 2022).

69 Urueña, *supra* note 5, at 283.

70 Massimiano Bucchi & Adrian Belton, *Beyond Technocracy: Democracy in the Age of Technoscience*, *in*Beyond Technocracy: Science, Politics and Citizens 73 (Massimiano Bucchi ed., 2009), https://doi.org/10.1007/978-0-387-89522-2_4 (last visited Feb. 23, 2023); Albert Meijer, Wouter Boon & Ellen Moors, *Stakeholder Engagement in Pharmaceutical Regulation: Connecting Technical Expertise and Lay Knowledge in Risk Monitoring*, 91 Public Adm. 696 (2013).

71 Guston, *supra* note 7, at 227.

72
Jonathan B. Wiener, Precautionary principle, https://www.elgaronline.com/display/book/9781785369520/b-9781785365669-VI_13.xml (last visited Feb. 23, 2023).

73 General Comment 25, paras. 56–57.

74
*Ibid*., paras. 56 (‘Technological and human rights impact assessments are tools that help to identify potential risks early in the process and the use of scientific applications’).

75 Iris Goldner Lang, *‘Laws of Fear’ in the EU: The Precautionary Principle and Public Health Restrictions to Free Movement of Persons in the Time of COVID-19*, Eur. J. Risk Regul. 1, 158 (2021) (with references to the jurisprudence of the European Court of Justice in public health cases); World Commission on the Ethics of Scientific Knowledge and Technology, *The Precautionary Principle*, (2005), https://unesdoc.unesco.org/ark:/48223/pf0000139578 (last visited Feb. 23, 2023) (‘the seriousness of the potential harm, consideration of their positive and negative consequences, and with an assessment of the moral implications of both actions and inaction’).

76 Monica van Hoof et al., *Factors Contributing to Best Practices for Patient Involvement in Pharmacovigilance in Europe: A Stakeholder Analysis*, 45 Drug Saf. 1083, 1084 (2022).

77
*Id.*

78 Christina Q. Nguyen et al., *The involvement of rare disease patient organisations in therapeutic innovation across rare paediatric neurological conditions: a narrative review*, 17 Orphanet J. Rare Dis. 167 (2022).

79 Andrea Boggio, ‘Invited Contributions: Responsible Anticipation’, *The GESDA 2023 Science Breakthrough Radar,* GESDA 2023, pp. 244–46, https://radar.gesda.global/trends/invited-contributions/responsible-anticipation (last visited Dec. 15, 2023).

80 General Comment 25, para 16.

81 Porsdam, *supra* note 47, at 6 (‘From a human rights perspective, scientific responsibility is the flip side of scientific freedom’). To be noted that the recognition of everyone’s right to responsible scientific progress does not mean that individuals have direct claims of scientific responsibilities against specific scientists. It means that individuals have an entitlement that their governments implement measures to ensure that researchers act responsibly.

82
*Ibid*., p. 24 (scientific responsibility is discussed in depth at pp. 94–96).

83 Roberto Andorno, *The Right to Science and the Evolution of Scientific Integrity*, *in*The Right to Science: Then and Now 91 (Helle Porsdam & Sebastian Porsdam Mann eds., 2021), https://www.cambridge.org/core/books/right-to-science/right-to-science-and-the-evolution-of-scientific-integrity/690D1992F0C0756FC2758FFCE5540262.

84 Heather Douglas, *Scientific Freedom and Social Responsibility*, *in*Science, Freedom, Democracy, 78 (Péter Hartl & Adam Tamas Tuboly eds., 2021).

85
*Id.*

86
*Id.*

87 Christophe Bernard, *Doing Socially Responsible Science in the Age of Selfies and Immediacy*, 9 eNeuro (2022), https://www.eneuro.org/content/9/2/ENEURO.0114-22.2022 (last visited June 22, 2022) (identifying ‘doing science, the validity of the discoveries themselves, and the consequences of these discoveries’ as the thee components of scientific responsibility).

88 Douglas, *supra* note 81, at 78.

89 Tamra Lysaght, *Anticipatory Governance and Foresight in Regulating for Uncertainty*, 22 Am. J. Bioeth. 51, 51 (2022).

90
*See*, UNESCO, *supra* note 39, para. 14(d)(v)(vi); 16(a)(ii); UNESCO, *supra* note 42, para I.1 (calling for ‘dealing responsibly with the known and unknown impacts of AI technologies on human beings, societies and the environment and ecosystems’).

91 UNESCO, *supra* note 39, para. 14(d)(v).

92
*Ibid.*, para. 14(d)(vi).

93
*Ibid.*, para. 16(a)(ii).

94 Paul Berg, *Asilomar 1975: DNA modification secured*, 455 Nature 290 (2008).

95 See, *Id.*

96
*Id.* at 290.

97 The Royal Society, *2023 Human Genome Editing Summit*, https://royalsociety.org/science-events-and-lectures/2023/03/2023-human-genome-editing-summit/ (last visited Feb. 23, 2023).

98
National Academies of Sciences, Engineering, and Medicine, Second International Summit on Human Genome Editing: Continuing the Global Discussion: Proceedings of a Workshop–in Brief (2019), https://doi.org/10.17226/25343 (last visited Feb. 23, 2023).

99 Statement from the Organising Committee of the Third International Summit on Human Genome Editing, https://royalsociety.org/-/media/events/2023/03/human-genome-editing-summit/statement-from-the-organising-committee-of-the-third-international-summit-on-human-genome-editing.pdf (last visiting Mar. 11, 2023).

100 See, National Academies, *On Human Gene Editing: International Summit Statement*, (2015), https://www.nationalacademies.org/news/2015/12/on-human-gene-editing-international-summit-statement (last visited Feb. 23, 2023).

101 Sheila Jasanoff, J. Benjamin Hurlbut & Krishanu Saha, *CRISPR democracy: Gene editing and the need for inclusive deliberation*, 32 Issues Sci. Technol. 25 (2015).

102 General Comment 25, para. 52.

103
*Ibid*., para. 10.

104 General Comment 25, para. 10 (‘The right enshrined in article 15 (1) (b) encompasses not only a right to *receive* the benefits of the applications of scientific progress, but also a right to *participate* in scientific progress. Thus, it is the right to participate in and to enjoy the benefits of scientific progress and its applications’) (emphasis added).

105 Lysaght, *supra* note 86, at 53.

106 Gene Rowe & Lynn J. Frewer, *Public Participation Methods: A Framework for Evaluation*, 25 Sci. Technol. Hum. Values 3, 254–255 (2000).

107
*Id.* at 255–256.

108 Eli Y. Adashi et al., *Heritable Human Genome Editing: The Public Engagement Imperative*, 3 CRISPR J. 434 (2020).

109 Advisory Committee on Developing Global Standards for Governance and Oversight of Human Genome Editing, *Call for contributions*, https://www.who.int/news-room/articles-detail/call-for-contributions-advisory-committee-on-developing-global-standards-for-governance-and-oversight-of-human-genome-editing (last visited Mar. 1, 2023).

110 Advisory Committee on Developing Global Standards for Governance and Oversight of Human Genome Editing, *Call for contributions: Printable Survey*, https://cdn.who.int/media/docs/default-source/ethics/printable-survey-july2020_e95068c5-1156-48b8-9316-76e3f5ac830c.pdf?sfvrsn=f8608ef0_26 (last visited Mar. 1, 2023).

111 Advisory Committee on Developing Global Standards for Governance and Oversight of Human Genome Editing, *Human Genome Editing: A DRAFT Framework for Governance*, (2020), https://cdn.who.int/media/docs/default-source/ethics/governance-framework-for-human-genome-editing-2ndonlineconsult.pdf?sfvrsn=524a0ebe_8 (last visited Mar. 1, 2023).

112
*Id.* at 21–40.

113 Donrich Thaldar et al., *A deliberative public engagement study on heritable human genome editing among South Africans: Study results*, 17 PLOS ONE e0275372 (2022).

114 Lluis Montoliu et al., *ARRIGE Arrives: Toward the Responsible Use of Genome Editing*, 1 CRISPR J. 128 (2018). ARRIGE was initiated by the French National Institute for Health and Medical Research (INSERM) Ethics Committee with four objectives: (1) fostering an inclusive debate with a risk-management approach, considering human, environmental, animal, and economic issues; (2) getting involved in the governance of genome-editing technology with governmental and intergovernmental stakeholders; (3)creating an ethical toolbox and informal guidance for genome-editing technology users, regulators, governance, and the civil society at large, including those living in low- and middle-income countries; and (4) developing a robust and specific reflection on the role of the lay public in this debate and the necessity for improved public engagement.

115 Sheila Jasanoff & J. Benjamin Hurlbut, *A global observatory for gene editing*, 555 Nature 435 (2018). The website is https://global-observatory.org/ (last visited Mar. 16, 2023).

116 John S. Dryzek et al., *Global citizen deliberation on genome editing*, 369 Science 1435 (2020).

117
*Id.* at 1435.

118
*Id.*

119 See, Samantha Besson, ‘The “human right to science” *qua* right to participate in science,’ The International Journal of Human Rights (2023), https://doi.org/10.1080/13642987.2023.2251897; Yvonne Donders & Monika Plozza, ‘Look before you leap: states’ prevention and anticipation duties under the right to science,’ The International Journal of Human Rights, https://doi.org/10.1080/13642987.2023.2269096; Rumiana Yotova, ‘Anticipatory duties under the human right to science and international biomedical law,’ The International Journal of Human Rights (2023), https://doi.org/10.1080/13642987.2023.2273289.

120 Andrea Boggio & Brian Gran, *A Proposal for Indicators of the Human Right to Science*, *in*The Right to Science: Then and Now 268 (Helle Porsdam & Sebastian Porsdam Mann eds., 2021), https://www.cambridge.org/core/books/right-to-science/9FC3AB86632A686C686F0C5FD8EBF766.

121 Jerome R. Ravetz, *The science of ‘what-if?,’* 29 Futures 533 (1997).

122 Rachel A. Ankeny, Megan J. Munsie & Joan Leach, *Developing a reflexive, anticipatory, and deliberative approach to unanticipated discoveries: Ethical lessons from iBlastoids*, 22 Am. J. Bioeth. 36, 42 (2022).

